# Case Report: Acupuncture is an effective treatment for olfactory dysfunction in the post COVID-19 condition

**DOI:** 10.3389/fneur.2022.916944

**Published:** 2022-08-23

**Authors:** Akira Morita, Aya Murakami, Takushu Uchihara, Noriyuki Ohashi, Koichi Ryu, Yuki Watanabe, Sadayuki Ochi, Kazuho Okudaira, Yoshiro Hirasaki, Takao Namiki

**Affiliations:** ^1^Department of Japanese-Oriental (Kampo) Medicine, Graduate School of Medicine, Chiba University, Chiba, Japan; ^2^Center for Pharmaceutical Education, Faculty of Pharmacy, Yokohama University of Pharmacy, Yokohama, Japan

**Keywords:** COVID-19, post COVID-19 condition, acupuncture, olfactory dysfunction, Yingxiang point (LI20)

## Abstract

Olfactory dysfunction in the post COVID-19 condition reported worldwide are refractory for some patients. For this reason, appropriate treatment is desired. In this article, we describe two cases of olfactory dysfunction in the post COVID-19 condition that was improved by traditional acupuncture treatment. By using the Yingxiang point (LI20), which is said to improve the sense of smell since ancient times, acupuncture treatment was performed 1–2 times a week in two patients about 6 and 7 months after the diagnosis of COVID-19. Acupuncture needles with a body length of 30 mm and a body diameter of 0.16 mm were inserted about 10 mm deep into the skin. We stimulated LI20 of the right and left sides until the patients felt the de qi sensation (acupuncture resonance), and left needles in the points for about 15 min. Immediately after the acupuncture treatment, the symptoms of olfactory dysfunction were alleviated, and the improvement in olfactory dysfunction lasted for 2–4 days. As the number of acupuncture treatments increased, the time until the flareup of olfactory dysfunction was prolonged, and the symptoms tended to decrease. In our experience, the acupuncture treatment was effective in a short period for treating residual olfactory dysfunction of the post COVID-19 condition, suggesting that acupuncture may serve as an adjunct to modern medical treatment, and it may also be a new option for patients who are resistant to Western medical treatment or unable to continue treatment because of side effects. In conclusion, acupuncture may be a new option for patients who are resistant to modern medical treatment or who are unable to continue treatment because of side effects.

## Introduction

Coronavirus disease (COVID-19) infections have been reported worldwide (1–6), and the World Health Organization defined the post COVID-19 condition as “symptoms that persist for more than 2 months after onset and cannot be explained by other diagnoses.” Although the characteristic olfactory disturbance due to COVID-19 (7) improves early over time in most cases, some patients remain symptomatic, which has a significant impact on their quality of life. In Japan, Miyazato et al. (5) reported that 16.1% of patients continued to have olfactory dysfunction at 2 months, 9.7% at 4 months, 7.7% at 6 months, and 1.1% at 12 months (6). In other geographically different countries, such as Italy, a study reported that 13.3% of patients still had olfactory dysfunction after about 110 days (8). Therefore, olfactory dysfunction after COVID-19 infection is a common disorder worldwide that is intractable in some patients.

Olfactory deficits are not only associated with reduced quality of life (e.g., loss of flavor in food and daily life), but also with life-threatening situations, such as food spoilage, gas leaks, and smoke from fires. The treatment of olfactory dysfunction depends on the cause, but known treatments include steroids, nasal spray, surgical treatment, and olfactory training (9–11), and other than these treatments, there are few effective treatments, and treatment options are limited (12, 13). Systemic steroids may promote the recovery of olfactory dysfunction in the post COVID-19 condition (14), but may not be continued because of resistance to these treatment or strong side effects. Efforts to alleviate and treat the post COVID-19 condition are eagerly awaited by many patients around the world.

The effects of acupuncture are being scientifically elucidated. It has been reported that skin stimulation is transmitted to the brain, activates the central nervous system (15, 16), and is involved in anti-inflammatory and immunomodulatory mechanisms (17, 18). We experienced two cases of improvement of olfactory dysfunction in the post COVID-19 condition after traditional acupuncture treatment. The Yingxiang point (LI20), a meridian point that has been said to improve the sense of smell since ancient times (19), was used, and it has been used to treat various nasal symptoms and facial paralysis. Because acupuncture treatment involving LI20 has been reported to improve olfactory dysfunction after the common cold (12, 20, 21), we performed acupuncture at the LI20 for olfactory dysfunction in the post COVID-19 condition. As far as we know, there is no case report of acupuncture treatment at LI20 reducing olfactory dysfunction in the post COVID-19 condition. These two cases we experienced may offer new options for olfactory dysfunction in the post COVID-19 condition; therefore, we report their treatment progress herein.

## Case descriptions

### Ethics statement

The patients provided written informed consent to participate in this case report and for the publication of any potentially identifiable images or data.

### Case 1

#### Clinical history

A 53-year-old woman was diagnosed with COVID-19 in May 2021 and was admitted to another hospital for 14 days. After discharge from the hospital, she still had some symptoms (malaise, olfactory dysfunction, taste dysfunction, midway awakening, decreased concentration, cough, shortness of breath on physical movement, insomnia, hair loss, etc.). Therefore, she was referred to our department in June 2021.

Table 1 shows the data of case 1 at the initial examination and at discharge. After the initial examination, treatment with Kampo prescription (Kamikihito) was started. There was a slight reduction in cough and general malaise, but there was no significant improvement. Hence, the patient was admitted to the hospital in October 2021. The level of olfactory dysfunction at admission was a numeric rating scale (NRS) score of 10 at the time of COVID-19 diagnosis, which persisted for approximately 5 months.

**Table 1:** Demographic and clinical characteristics of cases 1 and 2.

	**Case 1**		**Case 2**	
Age (year)	53		38	
Sex	F		M	
BMI (kg/m^2^)	26.3		36.1	
Medication history	Pregabalin		Acetaminophen	
	Mecobalamin			
	Theophylline			
	Omalizumab			
Medical history	Lower back pain		Tonsillectomy	
	Bronchial asthma			
**Blood test results**	**First visit**	**Discharge**	**First visit**	**Discharge**
TG (mg/dl)	127	68	223	195
LDL-cholesterol (mg/dl)	130	105	192	135
HDL-cholesterol (mg/dl)	59	60	65	53
HbA1c (NGSP, %)	5.4		5.5	
FPG (mg/dl)	135	99	95	125
SBP (mmHg)	139	125	149	143
DBP (mmHg)	78	80	95	103
CRP (mg/dl)	0.05	0.04	0.15	0.07
WBC (10^3^/μl)	4.2	4.8	10.8	7.3

*BMI, body mass index; F, female; M, male; TG, triglyceride; LDL, low-density lipoprotein; HbA1c, glycated hemoglobin A1c; FPG, fasting plasma glucose; SBP, systolic blood pressure; DBP, diastolic blood pressure; CRP, C-reactive protein; WBC, white blood cell*.

A subsequent venous olfactory test (alinamin test) result was unresponsive. An alinamin test is a test method that reflects olfactory mucosal disorders, in which prosultiamine is injected into a vein in 20 seconds (22). The time from the start of injection until the smell is perceived and the time until the smell disappears is measured. In cases of non-response to the alinamin test, a severe olfactory dysfunction involving the olfactory mucosa is suggested.

#### Acupuncture treatment

Acupuncture treatment was performed in October 2021 (about 5 months after the diagnosis of COVID-19). In the first session, the Shenmen point (HT7) was selected as the meridian point for the systemic symptom. After the second session, meridian points for systemic symptoms were the Yinlingquan point (SP9), Tianzhu point (BL10), Jueyinshu point (BL14), Xinshu point (BL15), Taixi point (KI3), Neiguan point (PC6), Daling point (PC7), Fengchi point (GB20), Taichong point (LR3), Danzhong point (CV7), Shengzhu point (GV12), and Baihui point (GV20). The acupuncture needle used for the systemic symptoms were 30–40 mm long and 0.14–0.18 mm in diameter, and they were inserted about 10 mm deep perpendicular to the skin.

**Figure 1:**
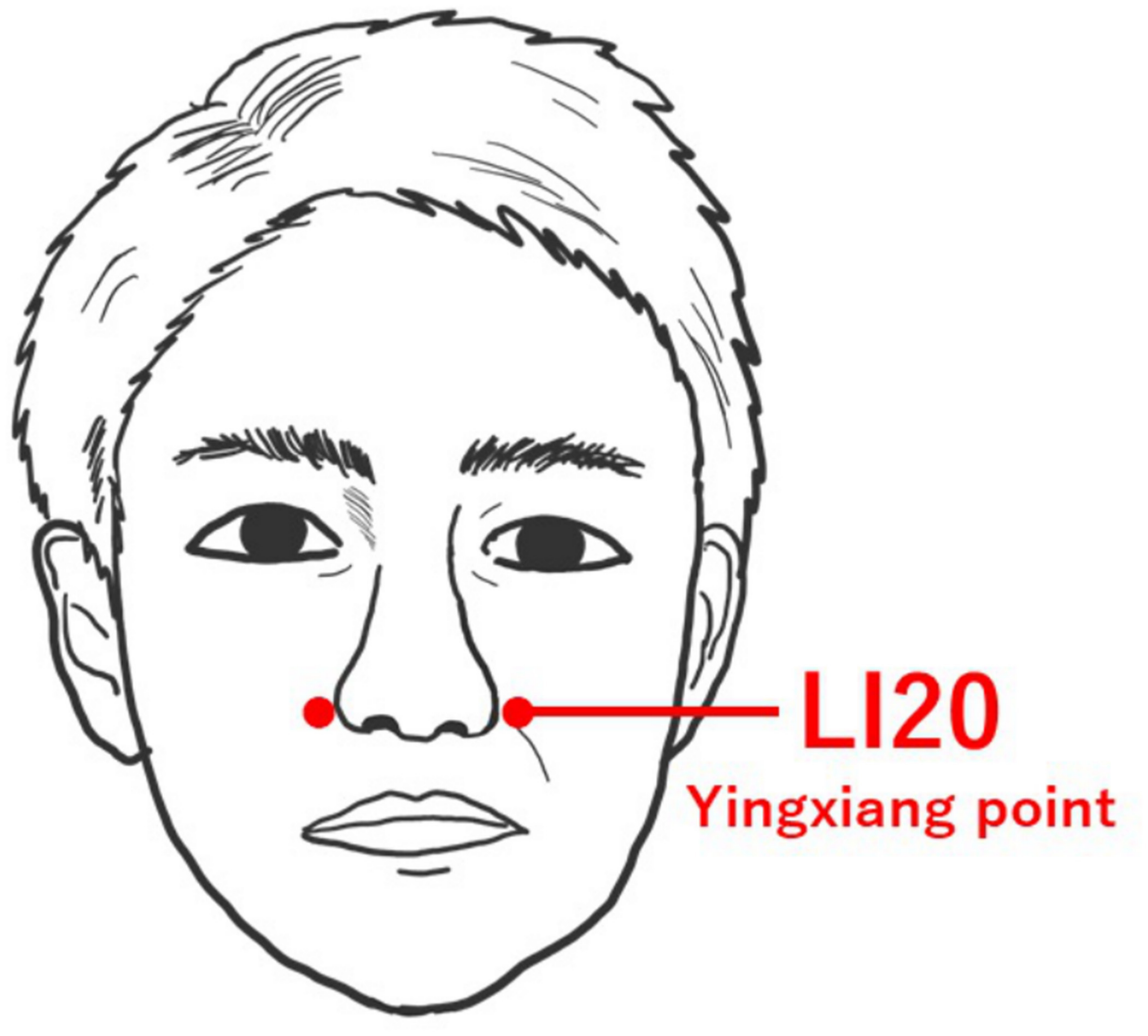
Location of LI20.

After the ninth session (November 16, 2021, which was performed 1 month later (approximately 6 months after the diagnosis of COVID-19), we added the LI20 of the right and left sides for olfactory dysfunction, which is located on the face, in the nasolabial sulcus, at the same level as the midpoint of the outer margin of the nasal wings (Figure 1). SP3 or LR3 were also used as meridian points for systemic symptoms. After the ninth acupuncture treatment, the acupuncture needles used for olfactory dysfunction were 30 mm long and 0.16 mm in diameter, and they were inserted about 10 mm deep into the skin. The acupuncture needles were stimulated until the de qi sensation (acupuncture resonance) was felt, and then the acupuncture needles were left in the points for 15 min. The de qi sensation is a special sensation felt by the patient and the acupuncturist during acupuncture, and it is thought that obtaining acupuncture resonance is closely related to the degree of the treatment effect (23).

Acupuncture treatment was performed twice a week during hospitalization, and was performed once a week after discharge.

#### Treatment response

**Figure 2:**
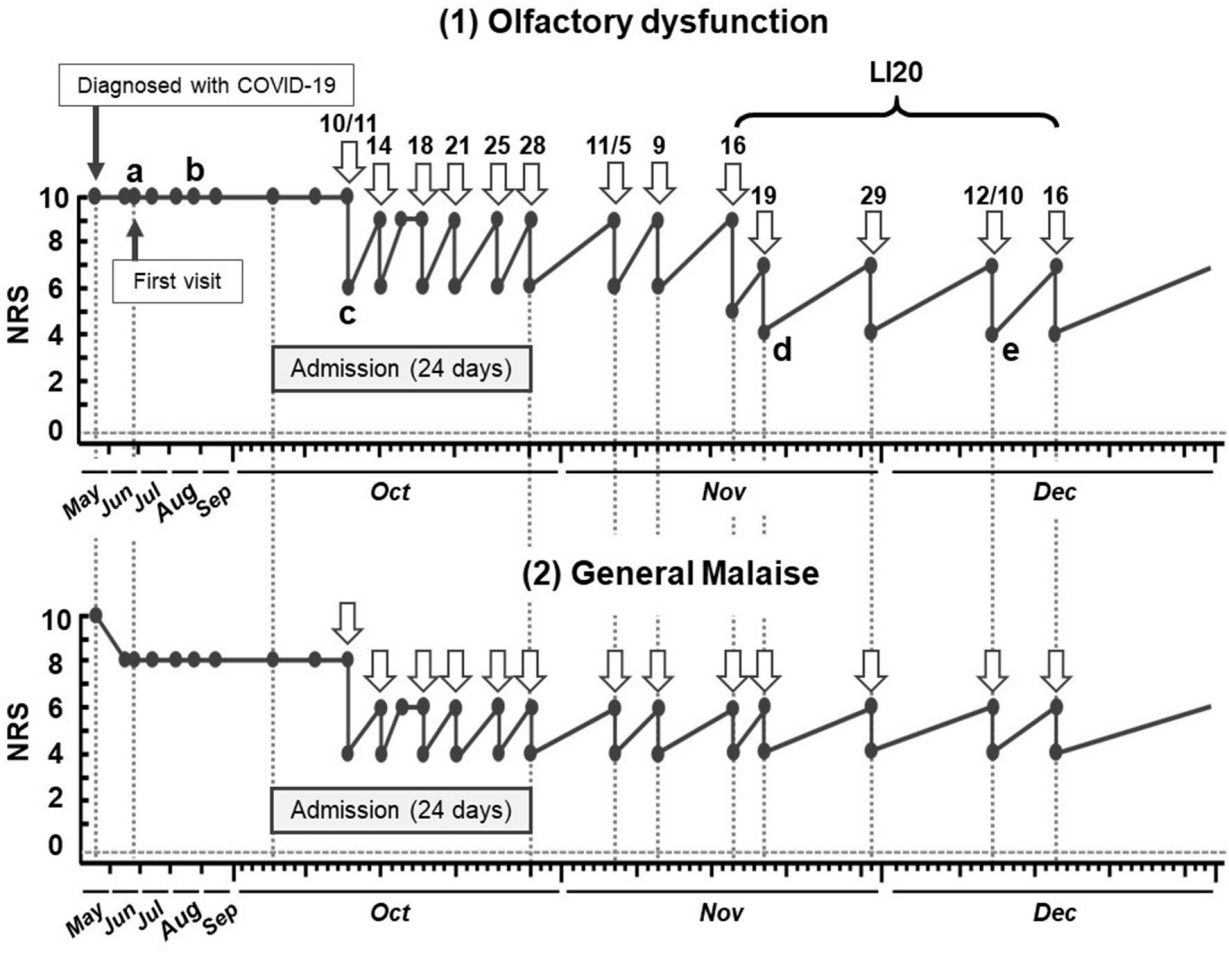
Timeline of acupuncture treatment and subjective symptoms of olfactory dysfunction and general malaise in case 1. a. Difficult to smell. b. Cannot smell chlorine bleach (for cleaning) and does not feel well. c. Recognizes slight smell of sewage and the smell of coffee. d. Can smell own stool and ginger tea. e. Can smell sewage and herbs after treatment. NRS, numeric rating scale; COVID-19, coronavirus disease.

The degree of the patient's olfactory dysfunction was evaluated with the Numeric Rating Scale (NRS). The NRS is an evaluation method that expresses subjective sensations, such as pain, stress, and daily life obstacles that the patient is aware of, as an objective numerical value to share with others. In response to the question, “If the level of olfaction before infection was 0 and the level of not smelling at all was 10, how much is it now?” patients were asked to express their current subjective symptoms on an 11-point scale from 0 to 10. The timeline of the acupuncture treatment and NRS for olfactory dysfunction and general malaise are shown in Figure 2. After the first session, the patient returned to her hospital room and became aware of the smell of coffee and slight smell of sewage. This was the first relief of olfactory dysfunction that had remained constant for about 5 months (the NRS score decreased from 10 to 6 of immediately after acupuncture treatment). Similarly, relief of general malaise was observed immediately after treatment. The reduction in both olfactory dysfunction and general malaise were maintained for 2–3 days, but then flared up to the pre-acupuncture level. After six sessions (twice a week) were given during the hospitalization period, the patient switched to outpatient treatment because she wished to continue treatment with Kampo and acupuncture. However, after a total of eight sessions including both during hospitalization and after discharge, the patient still sustained an NRS score of 9.

When LI20 was added, starting from the ninth sessions (approximately 6 months after the diagnosis of COVID-19), the NRS score was reduced to 4 immediately after the treatment, and it was maintained at 7 after 2–3 days. After discharge from the hospital, the patient was treated once a week.

No adverse reactions were observed throughout the treatment period, including outpatient treatments. Regarding the Kampo prescription, we continued to use Kamikihito, which she had been taking since her admission.

### Case 2

#### Clinical history

A 38-year-old man was diagnosed with COVID-19 in May 2021. He was hospitalized for 8 days until May 25, 2021 and received oxygen inhalation and other therapeutic measures, but some symptoms (malaise, olfactory dysfunction, midway awakening, decreased concentration, palpitations, fatigue after physical activity, heaviness in the head, etc.) remained after discharge, and he was referred to our department in July 2021.

Table 1 shows the information of case 2 at the initial examination and discharge. After starting Kampo prescription (Ninjin'yoeito), there was a tendency for fatigue after physical activity and olfactory dysfunction to be reduced, but the symptoms flared up again. After the symptoms continued for another 30 days, the patient was admitted to the Department of Kampo Medicine in November 2021 (about 6 months after the diagnosis of COVID-19). After admission, the patient remained unchanged for another 40 days with olfactory dysfunction (NRS score, 3) and general malaise (NRS score, 5).

#### Acupuncture treatment

In December 2021 (approximately 7 months after the diagnosis of COVID-19), the first acupuncture treatment was performed for systemic symptoms and olfactory dysfunction. Case 2 used LI20 from the first treatment. The acupuncture points, needles used, insertion depth, and stimulation method were the same as those in case 1. The acupuncture treatments from the second time onward were performed in the same way as the first treatment, and once a week during hospitalization. After discharge from the hospital, treatment was given twice a week to maintain the effect.

#### Treatment response

**Figure 3:**
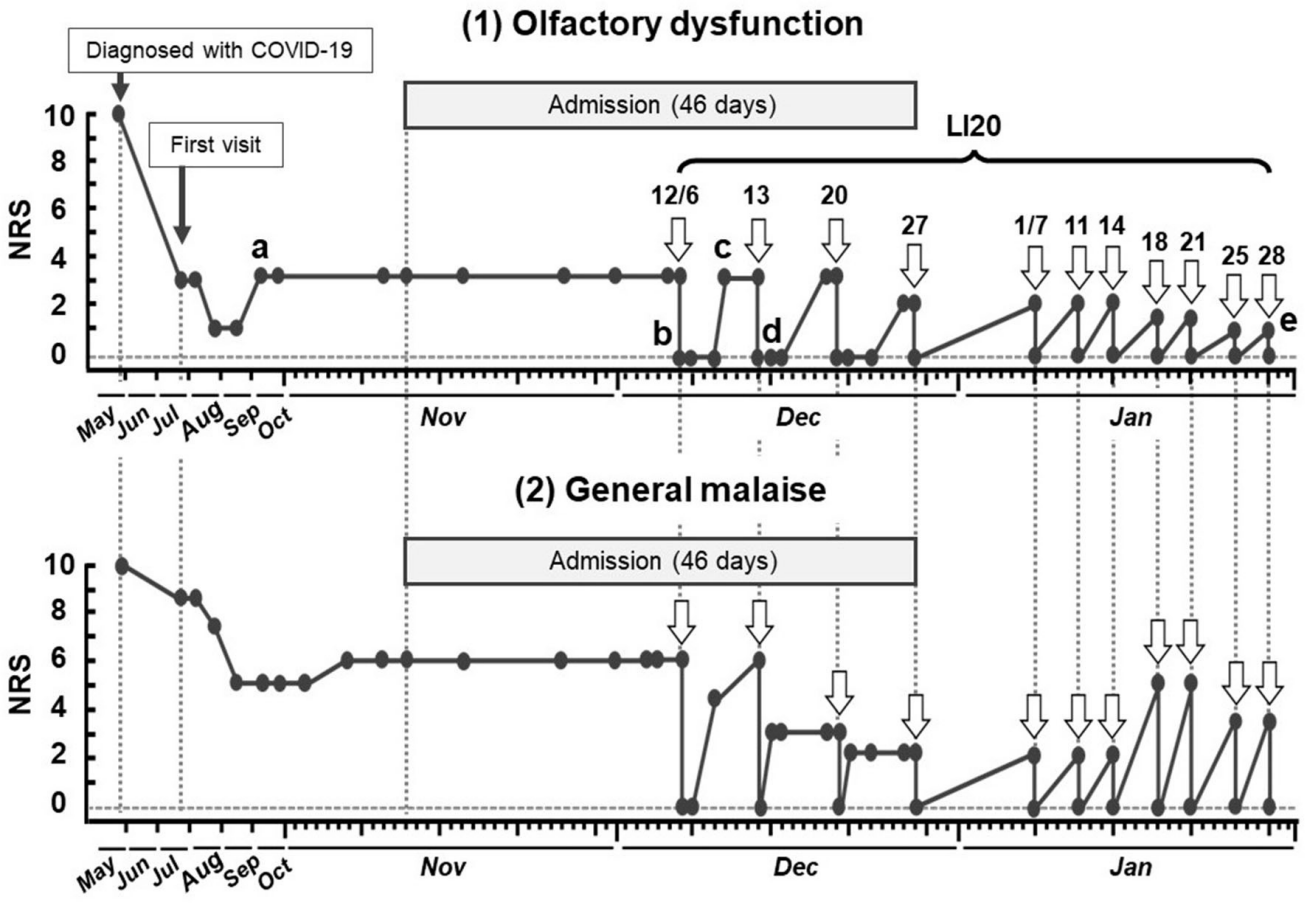
Timeline of acupuncture treatment and subjective symptoms of olfactory dysfunction and general malaise in case 2. a. Cannot recognize the smell of coffee. b. Recognizes the smell of alcohol, mayonnaise, and soy sauce in the room. c. Cannot recognize the smell of coffee. d. Can smell toothpaste. e. The smell of coffee is noticeable. NRS, numeric rating scale; COVID-19, coronavirus disease.

The subjective symptoms of olfactory dysfunction and general malaise are shown in Figure 3. After the first acupuncture treatment, the patient returned to the hospital room and became aware of the smell of alcohol in the room. The improvement of olfactory dysfunction lasted for 3–4 days, after which it flared up to the pre-treatment symptom level (NRS score, 3). After the second and third acupuncture treatments, as after the first treatment, there was a rapid reduction in symptoms (NRS score, 0). As the number of acupuncture treatments increased, the time until the flareup of olfactory dysfunction was slightly prolonged, and the level of symptoms tended to decrease.

A total of four acupuncture treatments (once a week) were given during the hospitalization period. After discharge, acupuncture treatments were continued twice a week. No adverse reactions were observed during the treatment period, including outpatient treatment. Regarding the Kampo prescription, he continued to take Ninjin'yoeito from the time of admission.

## Discussion

The cases described herein suggest that acupuncture may have a positive effect on olfactory dysfunction in the post COVID-19 condition.

LI20 is located in the region of the trigeminal nerve (second branch), which transmits perception to the brain. The branches of the second branch (maxillary nerve) become mixed nerves with sympathetic and parasympathetic nerves (i.e., posterior nasal nerves) and reach the nasal cavity. Previous reports on acupuncture for allergic rhinitis suggest that it may modulate anti-inflammatory effects including neural pathways, involve the down-regulation of various cytokines (17, 18), and some reports suggest improved nasal ventilation due to sympathetic nerve dominance (24). The mechanism by which the reduction in olfactory dysfunction was achieved in this case is unknown, but previous reports showed that acupuncture may have had an effect that included anti-inflammatory effects and nerve activation. Moreover, a previous study of olfactory abnormalities after upper respiratory tract infection considered that acupuncture with LI20 may have a positive effect on the cognitive processing of odors (12). Therefore, the same possibility is considered for olfactory dysfunction in the post COVID-19 condition.

Acupuncture treatment also alleviated malaise in our cases. This may be because of the positive effects of acupuncture stimulation of the upper and lower extremities at the Shenmen (HT7), Taibai (SP3), or Taichong (LR3) points on the visceral organs, especially the digestive system, through supraspinal reflexes (25, 26). In addition, one of acupuncture effects includes relaxation (27, 28), and the fact that both patients were aware that they did not wake up in the middle of the night after the acupuncture treatment suggests that it had a positive effect on their mind and body. On the next day after treatment, malaise flared up, and compared to the reduction of olfactory dysfunction, which lasted for about 3 days, the continuous effect was lower. This may be due to the fact that the acupuncture treatment for local stimulation was focused on only olfactory dysfunction.

In case 2, the patient's olfactory dysfunction eased to a level where he was not aware of any discomfort (NRS score, 0–1). However, in case 1, the NRS was only decreased to 7. One factor for this could be the frequency of treatment with LI20: case 2 continued to be treated twice a week, whereas case 1 was treated once a week. Some reports indicate that acupuncture treatment three times a week was more effective in analgesia than acupuncture treatment once a week (29). Therefore, it can be inferred that treatment frequency and efficacy may be related in the improvement of olfactory function as well.

Higher treatment frequency with LI20 can be considered to lead to higher treatment efficacy. An additional factor could be the difference in the level of damage to the olfactory mucosa cells.

Regarding the mechanism of olfactory deficits after COVID-19 infection of olfactory epithelial support cells, it has been suggested that severe acute respiratory syndrome coronavirus 2 (SARS-CoV-2) may cause damage to the nasal mucosa *via* inflammation (30). Furthermore, experiments with hamsters revealed the shedding of the olfactory epithelium, which contains olfactory receptors (receptors that receive odorants), early after infection (31). In general, the olfactory epithelium regenerates and returns to normal thickness after injury. However, in the case of SARS-CoV-2 infection, it has been reported that the degree of injury and the speed of regeneration vary depending on the site, with most of the epithelium returning to normal thickness and some remaining damaged. Considering these SARS-CoV-2-specific symptoms, the results of the alinamin test, and NRS changes from the time of diagnosis to the first visit, we speculate that the level of damage to the olfactory mucosa in case 1 may have been stronger than that in case 2. Perhaps further symptom relief could have been obtained with high frequency acupuncture treatments with LI20.

Lastly, we will discuss the limitations of this case report. First, we have not been able to identify the type of variant that affected these patients. Based on the time when these two cases were obtained, it is expected to be the post COVID-19 condition caused by the delta variant that was spreading in Japan, but we would like to examine the effect of different variants. Second, these patients were treated with Kampo prescriptions too, so the effect of acupuncture alone could not be determined. Kampo prescription has the effect of enhancing the body's natural healing power. Kamikihito, prescribed in case 1, consists of 14 crude drugs (32) and is a Kampo prescription known to be effective in treating insomnia, anorexia, and depression (33). Ninjin'yoeito, prescribed in case 2, consists of 12 crude drugs and is used for recovery from decreased physical strength after illness or surgery and improvement of symptoms such as fatigue, anorexia (34). In our clinical practice, we have experienced the synergistic effect of acupuncture and Kampo prescription together, and in these cases, the Kampo prescription was able to regulate the digestive symptoms and correct the distortion of the biological balance, which was considered to be an important factor that enhanced the original effect of acupuncture. The third factor was weight control during the hospitalization. Obesity has been pointed out as one of the factors that prolong the post COVID-19 condition (35), and both of the patients were obese. Partly due to calorie control during hospitalization, case 1 lost 3 kg and case 2 lost 9 kg from the time of initial examination to the time of discharge. Although it is not possible to clearly conclude the effect of weight loss, this case study reduced symptoms in patients with such a high body mass index, regardless of sex or age.

Despite these limitations, our experiences show that acupuncture treatment can be an adjunct to modern medical treatment as it is effective within a short period for treating residual olfactory dysfunction after healing from COVID-19 infection. The application of acupuncture treatment may be a new option for patients who are resistant to modern medical treatments or who are unable to continue treatment because of strong side effects.

## Data availability statement

The raw data supporting the conclusions of this article will be made available by the authors, without undue reservation.

## Ethics statement

Ethical review and approval was not required for the study on human participants in accordance with the local legislation and institutional requirements. The patients/participants provided their written informed consent to participate in this study. Written informed consent was obtained from the individual(s) for the publication of any potentially identifiable images or data included in this article.

## Author contributions

AMo and TU conducted the acupuncture treatment with support from NO and KR. AMo and AMu wrote the manuscript with support from YW, SO, KO, YH, and TN. AMu analyzed the data with support from TN. AMo and TN conceived the original idea. TN supervised the project. All authors discussed the results and contributed to the final manuscript.

## Funding

The authors are extremely grateful to the Japan Agency for Medical Research and Development (AMED) for providing funding (Grant No: JP211k0310078) in this study.

## Conflict of interest

The authors declare that the research was conducted in the absence of any commercial or financial relationships that could be construed as a potential conflict of interest.

## Publisher's note

All claims expressed in this article are solely those of the authors and do not necessarily represent those of their affiliated organizations, or those of the publisher, the editors and the reviewers. Any product that may be evaluated in this article, or claim that may be made by its manufacturer, is not guaranteed or endorsed by the publisher.
